# P-146. Individual-level factors associated with acute hospitalization after medically attended acute gastroenteritis and norovirus gastroenteritis in the United States, 2022–2024

**DOI:** 10.1093/ofid/ofaf695.372

**Published:** 2026-01-11

**Authors:** Christine Kim, John Shen, Wen-Hsing Wu, Elissa Wilker, Brandon J Patterson, Christopher Bush, Ben Lopman, Daniel C Payne, Evan J Anderson, Katherine B Carlson

**Affiliations:** Moderna, Inc., Manassas, VA; Aetion, Boston, Massachusetts; Moderna, Inc., Manassas, VA; Moderna, Cambridge, Massachusetts; Moderna, Inc., Manassas, VA; Aetion, Inc., New York, City, New York; Rollins School of Public Health, Emory University, Atlanta, GA; Cincinnati Children's Hospital Medical Center, Decatur, GA 30030-3637, Georgia; Moderna, Inc., Manassas, VA; Moderna, Cambridge, Massachusetts

## Abstract

**Background:**

Norovirus gastroenteritis (NGE) is the leading cause of acute gastroenteritis (AGE) in the US but often undiagnosed due to limited clinical testing. Underlying medical conditions such as immunocompromising disorders increase the risk of severe outcomes from AGE and NGE and may exacerbate pre-existing conditions, worsening clinical outcomes. In this analysis, we estimated the association between underlying medical conditions and acute all-cause hospitalization among patients with medically attended AGE (MA-AGE) or MA-NGE.

**Figure:**
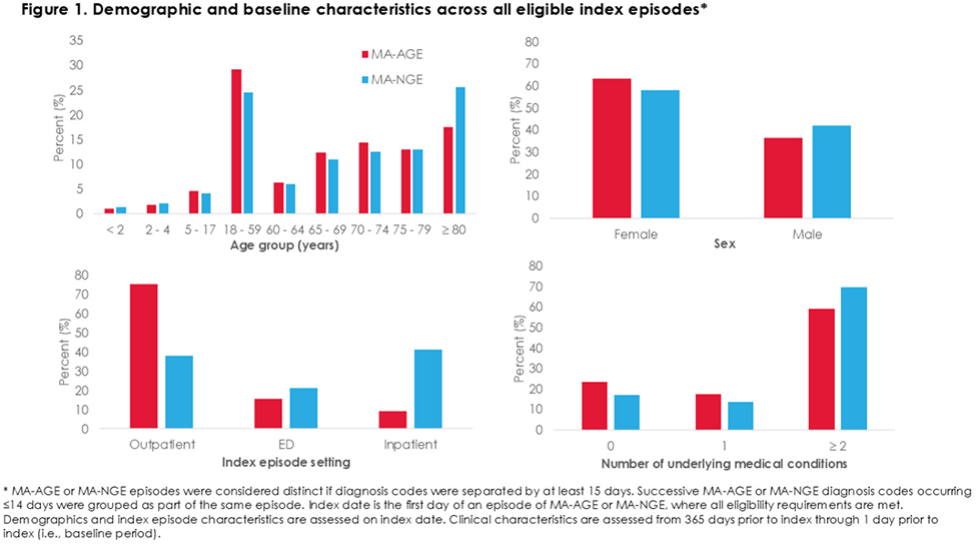

**Figure:**
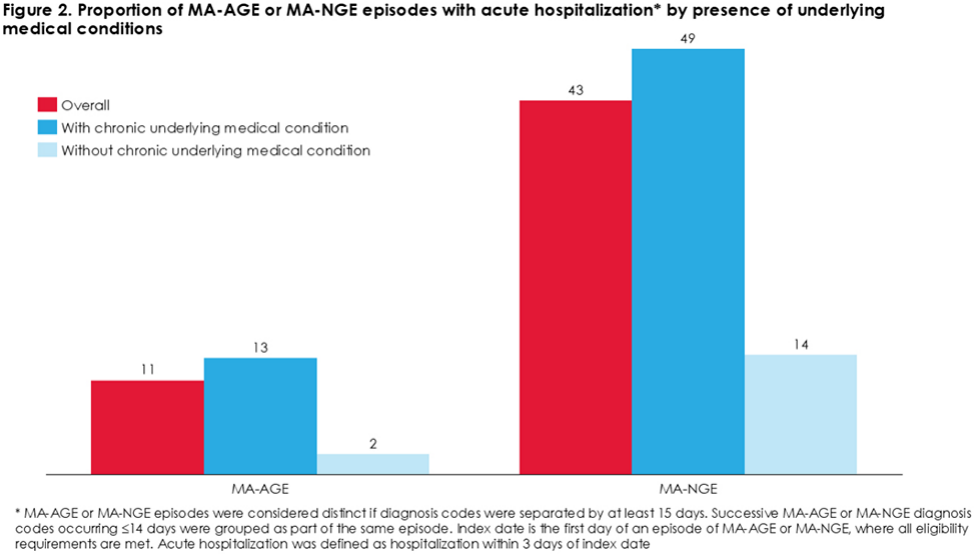

**Methods:**

We conducted a retrospective cohort study using Optum’s de-identified Clinformatics® Data Mart Database (Optum® CDM) of commercially insured and Medicare Advantage members. MA-AGE and MA-NGE episodes ≥ 14 days apart were identified via ICD-10 codes from July 1, 2022–June 30, 2024. Demographics and underlying medical conditions were identified in the year prior to index. Acute hospitalization was assessed within 3 days of index date. Generalized estimating equations adjusted for age, sex, and US Census region were used to calculate adjusted risk ratios (aRR) and 95% confidence intervals (95% CI) for associations with acute hospitalization.

**Figure:**
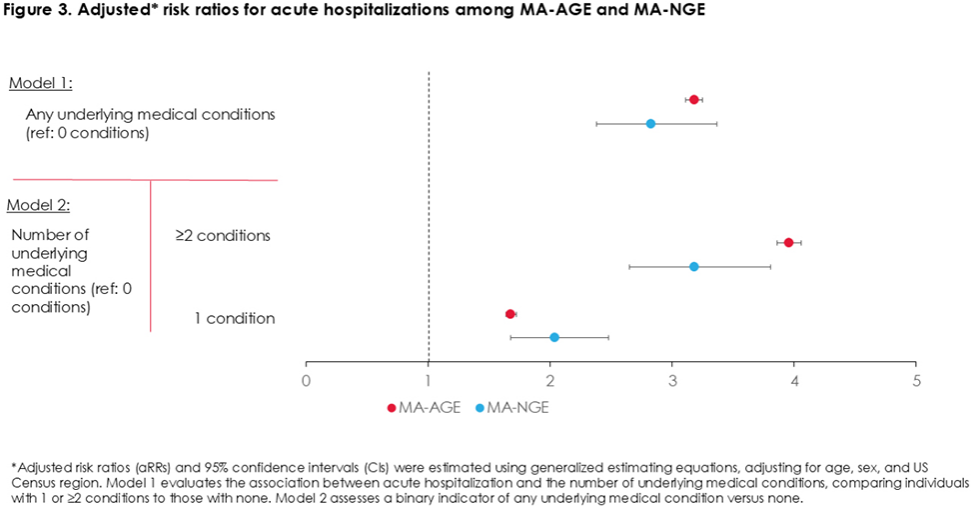

**Results:**

We identified 1,785,834 and 6,252 MA-AGE and MA-NGE episodes, respectively. Over 60% occurred in adults ≥ 60 years and over 75% in individuals with ≥ 1 underlying condition (Figure 1). Acute hospitalization occurred in 11% of MA-AGE and 43% of MA-NGE episodes (Figure 2). Compared to those without underlying medical conditions, having ≥ 1 condition was associated with greater acute hospitalization risk [MA-AGE aRR (95% CI): 3.18 (3.11-3.23); MA-NGE: 2.83 (2.38-3.37)] (Figure 3, model 1). Risk further increased for individuals with ≥ 2 conditions (Figure 3, model 2).

**Conclusion:**

Underlying medical conditions were strongly associated with higher risk of acute hospitalization for AGE and NGE, even after adjusting for known risk factors including age and sex. These findings align with prior research and emphasize the need for enhanced prevention strategies, including vaccines, particularly for individuals with underlying medical conditions. Future work may explore analytic methods to account for underreporting of NGE cases in real-world data given diagnostic limitations.

**Disclosures:**

Christine Kim, PhD, MSPH, Director, Moderna, Inc.: Employee|Director, Moderna, Inc.: Stocks/Bonds (Public Company) John Shen, MSPH in Epidemiology, Moderna: Contracted Research Wen-Hsing Wu, MS, Moderna: Stocks/Bonds (Public Company) Elissa Wilker, ScD, AstraZeneca: Stocks/Bonds (Public Company)|Moderna: Salary|Moderna: Stocks/Bonds (Public Company) Brandon J. Patterson, PharmD, PhD, Moderna: Employment|Moderna: Stocks/Bonds (Private Company) Christopher Bush, MPH, Aetion, Inc.: Stocks/Bonds (Private Company) Ben Lopman, PhD, Epidemiological Research and Methods: Advisor/Consultant|Hillevax: Advisor/Consultant Daniel C. Payne, PhD, MSPH, Merck: Advisor/Consultant|Moderna: Advisor/Consultant Evan J. Anderson, MD, Moderna: Stocks/Bonds (Public Company) Katherine B. Carlson, PhD, MPH, Moderna: Employee|Moderna: Stocks/Bonds (Private Company)

